# Effect of the Walking Exercise Program on Cancer-Related Fatigue in Patients with Acute Myeloid Leukemia Undergoing Chemotherapy

**DOI:** 10.31557/APJCP.2019.20.6.1661

**Published:** 2019

**Authors:** Fatemeh Gheyasi, Shahram Baraz, Amal Saki Malehi, Ahmad Ahmadzadeh, Reza Salehi, Mojtaba Vaismoradi

**Affiliations:** 1 *Nursing Care Research Center in Chronic Diseases, *; 2 *Thalassemia and Hemoglobinopathy Research Center, Research Institute of Health, *; 3 *School of Rehabilitation, Ahvaz Jundishapur University of Medical Sciences, Ahvaz, Iran, *; 4 *Faculty of Nursing and Health Sciences, Nord University, Bodø, Norway. *

**Keywords:** Acute myeloid leukemia, cancer-related fatigue, aerobic exercise, walking program

## Abstract

**Background:**

Cancer-related Fatigue (CRF) is one of the most common complications of acute myeloid leukemia (AML) and its related therapies. It can influence all physical and psychological aspects of the patient’s life. Also, it is believed that exercise can improve CRF in patients with cancer.

**Objective:**

This study aimed to investigate the effect of the walking exercise program on CRF in patients with AML undergoing chemotherapy.

**Methods:**

In this quasi-experimental study with a pre- and post-test design, 50 patients with AML undergoing chemotherapy were selected using a convenience sampling method at a teaching hospital in an urban area of Iran. The intervention included daily 30 minutes of planned walking for ten days. Data was collected using a demographic data form and the Brief Fatigue Inventory, which were filled out before the intervention, and on the fifth and tenth days of the intervention.

**Findings:**

Statistically significant differences were reported in the reduction of CRF on the fifth day and tenth day of the intervention (p <0.001).

**Conclusions:**

The planned walking intervention can be used as an easy and low-cost method for reducing CRF in patients with leukemia.

## Introduction

According to the National Cancer Institute (2013), Acute Myeloid Leukemia (AML) is developed from myeloid progenitors with abnormal functions. AML as a heterogeneous disease is characterized by various genetic abnormalities and prognoses (Smith-Turchyn AND Richardson, 2015). One of the standard procedures for the treatment of AML is the utilization of chemotherapy followed by bone marrow transplantation (Battaglini et al., 2009). It is believed that treatment can improve the overall patients’ survival rate, but it can endanger physical and psychological functions and the quality of life of patients (Dennett et al., 2016; Miladinia et al., 2017). 

Cancer-related fatigue (CRF) is one of the most disturbing complaints of patients suffering from AML and is reported in the majority of patients during chemotherapy (Miladinia et al., 2018; Mustian et al., 2012). CRF is defined as a distressing, persistent, subjective sense of physical, emotional and/or cognitive tiredness or exhaustion related to cancer and cancer treatment. It can interfere with the activities of daily living (Mustian et al., 2012). Many patients report the experience of CRF for months or years even after the successful treatment (Meneses-Echavez et al., 2015). No appropriate drugs for the prevention or treatment of CRF has been suggested, but recent systematic reviews indicated that physical activity and aerobic exercise interventions have been helpful for the reduction of CRF and improvement of patients’ quality of life (Dennett et al., 2016; Tian et al., 2016; Brown et al., 2010). Aerobic exercise activities including walking, biking, jogging, swimming, aerobic exercise and cross-country skiing have been suggested (Tian et al., 2016). 

Walking is a simple exercise and more accessible and adaptable than other type of exercise (Tsianakas et al., 2017). An early study by Chang et al., (2008) showed a lower level of CRF in patients with AML undergoing chemotherapy during a three-week walking exercise program. Wenzel et al., (2013) also found that home-based walking was associated with less CRF and less emotional distress. On the other hand, Zou et al., (2014) reported contradictory results and demonstrated that aerobic exercise had no significant effects on the Functional Assessment of Chronic Illness Treatment-Fatigue (FACIT-F) between intervention and control groups in patients with breast cancer. 

*Background of nursing in Iran*

One of the caring challenges in Iranian nursing is insufficient knowledge and skills of healthcare staff about the use of exercise programs for relieving CRF among patients suffering from cancer. Routine care delivered to patients with cancer undergoing chemotherapy consists of usual recommendations and encouragements for exercise by an oncologist or nurse in clinical settings. Moreover, a few studies have been conducted on the use of exercise programs in patients with cancer in Iranian healthcare settings. Therefore, there is a need to further research with the aim of improving nurses’ knowledge about how to use such programs and improve patients’ health conditions. To cover this gap, the aim of this study was to investigate the effect of the walking exercise program on CRF in patients with AML undergoing chemotherapy in an urban area of Iran. 

## Materials and Methods

*Study design*

This was a quasi-experimental study with a pre- and post-test design. The reason for the selection of this pragmatic research design rather than a two-group randomized design was the low number of sample size in the research zone.

*Sample and setting*

A sample size of 50 patients was estimated using a formula given an alpha 0.05, power 80% and a possible attrition rate of 10%. This study was carried out at a referral teaching hospital affiliated with Ahvaz Jundishapur University of Medical Sciences and as the only specialized cancer and chemotherapy hospital in the south west of Iran from December 2016 to July 2017. All eligible patients referred to the hospital were selected using a convenience sampling method. 

Inclusion criteria for the recruitment of the subjects were (i) age 18 years and older; (ii) being diagnosed with acute AML or the recurrence of AML; (iii) taking part in at least two chemotherapy sessions; (iv) no history of underlying diseases including cardiovascular diseases, diabetes mellitus and hypertension; (v) having no other types of cancer at the time of this study. Exclusion criteria were an inability to attend two walking sessions, hemodynamic instability, discharge from the hospital or death, life expectancy less than one month, and intractable or uncontrolled pain. It was noted that the chemotherapy treatment regimen for almost all patients with AML was consisted of Cytarabine and Daunorubicin.

*Ethical considerations*

This study was approved by the Ethics Committee affiliated with the University of Medical Sciences in which the second author worked (decree code: IR.AJUMS.REC.1395.663). Ethical considerations were in accordance with the Helsinki Declaration 1995, revised 2001. The aim and method of the study were explained to the patients and their questions were answered by the first researcher. They were also assured that the intervention had no consequences on the patients’ health and did not interfere with their treatment process. They could withdraw from the study at any time without any effect on their caring process. The written informed consent form was signed by those patients who willingly agreed to take part in this study. Their confidentiality and anonymity were ensured throughout the study process. This study was registered in the Iranian website of Registry of Clinical Trial under the code of IRCT20091017002599N2.

*Data collection*

Data was collected using the demographic data form and the Brief Fatigue Inventory (BFI), which were filled out by the first author before the intervention, and on the fifth and tenth days of the intervention. The demographic data form was consisted of questions about age, gender, the marital status, education level, occupation status, duration of disease, and frequency of chemotherapy. It was completed before the intervention through interviewing the patients and reviewing their medical files. 

*Fatigue intensity and interference*

The fatigue experience consisting of fatigue intensity and interference with daily life activities was assessed using the BFI. It is a reliable tool for measuring CRF in patients with cancer (Mendoza et al., 1999). BFI contains nine questions. The first three questions examine the intensity of fatigue in the last 24 hours such as the current level of fatigue, usual level of fatigue in the last 24 hours, and the worst level of fatigue in the last 24 hours. Also, the next six questions assess fatigue interference with daily life activities during the past 24 hours such as interference with general activities, mood, normal work, walking ability, relations with other peoples and enjoyment of life. Responses were scored from 0 to 10 with 0 indicating no fatigue (or no interference) at all and 10 indicating the worst fatigue or interference with daily life activities. Also the mean score of 6 questions on fatigue interference with daily life activities was used to show the impact of fatigue on the patients’ daily life. Reliability of the instrument was confirmed by Chang et al., (2008) using the calculation of the Cronbach’s alpha coefficient (r=0.9). In this study, reliability of the instrument also was confirmed (r=0.89). 

*Intervention*

*Theoretical background of the study*

In this study, a walking program based on the physical activity intervention was designed for patients with AML undergoing chemotherapy. This program aimed to relieve CRF based on the Bandura Self-Efficacy Theory (BSET) (Bandura, 1997). Bandura believes that after performing self-efficacy interventions to improve patients’ self-efficacy beliefs, they are encouraged to do exercise and achieve health-related outcomes. Once self-efficacy beliefs are established, actions are taken to reach desired outcomes. The BSET consisting of ‘individual’, ‘efficacy beliefs’, ‘behavior’, ‘outcome expectations’ and ‘outcomes’ is developed based on achieving optimal results through the use of different methods for reinforcing self-efficacy beliefs (Bandura, 1997). The determinants of self-efficacy beliefs are ‘enactive mastery experiences’, ‘verbal persuasion’, ‘vicarious experiences’ and ‘emotional/physical arousal’. The sources of outcome expectations flow through a specified pathway and create positive and negative effects on physical, social, and self-evaluation dimensions (Bandura, 1997a,b). 

*Intervention*

After choosing the eligible patients and before the intervention, the demographic data form and the BFI were filled out. The researcher provided a quiet and private place in the hospital’s courtyard to perform the walking exercise program. Before each session, the patients’ vital signs including blood pressure, pulse and respiratory rates were measured by the first researcher. The patients were assigned to the group of males and females to facilitate the implementation of the walking exercise program in each gender group. Similar to the intervention by Wenzel et al., (2013), the patients were asked to perform a 30-minute walk program in two 5-day non-stop periods and within a specified time each day in the following phases: warm-up and preparing the body (5 minutes), a fast-paced walking on the basis of their tolerance (10 minutes), slow-paced walking and body cooling (5 minutes) followed by 10 minutes of rest and relaxation. 

The patients walked slowly in the warm-up phase and took deep breath and then entered the main walking program including a 10-minute quick-paced walking. Subsequently, the patients were asked to walk in a slow pace to feel their heartbeat and do cool down exercises for 5 minutes. After the walking exercise program, the patient laid on the bed and breathed deeply for ten minutes, and their vital signs were measured again by the researcher. On the fifth and tenth days of the intervention, the BFI was completed again. 

In this study, the BEST was used in terms of the use of (a) heart rate monitor loop as the patients were taught to put the loop on their index finger when walking; (b) pedometer; (C) making phone calls to the researcher to report their exercise process every other day; (d) taking sports notes every three days; (e) holding a session every three days with the researcher and (f) being a role model that encouraged the patient’s self-efficacy and emphasized the acceptance of the walking program by the patients (Table 1).

The severity of the walking exercise program was mild to moderate and based on 40-60% of the maximum heart rate. The maximum heart rate was measured by the heart rate control loop at the end of the fast walking phase. The maximum heart rate was used to determine the suitability of the severity of walking for each patient (Wang et al., 2011). The walking program’s fidelity was assessed by making telephone calls to the patients, daily self-monitoring during the walking program and holding sessions with the patients by the researcher.

*Data Analysis*

Data analysis was performed using descriptive and inferential statistics via the SPSS software v.22. The mean, standard deviation, relative frequency, absolute frequency and repeated measures ANOVA test were used to report findings. P < 0.05 was set as statistically significant.

## Results

The patients were mostly male (60%), married (50%), had an undergraduate education level (32%) and were employees (46%). The mean duration of AML, mean age of the patients, and average number of chemotherapy sessions were 34 weeks, 37±12.3 years and 5 sessions, respectively (Table 2). 

**Figure 1:**
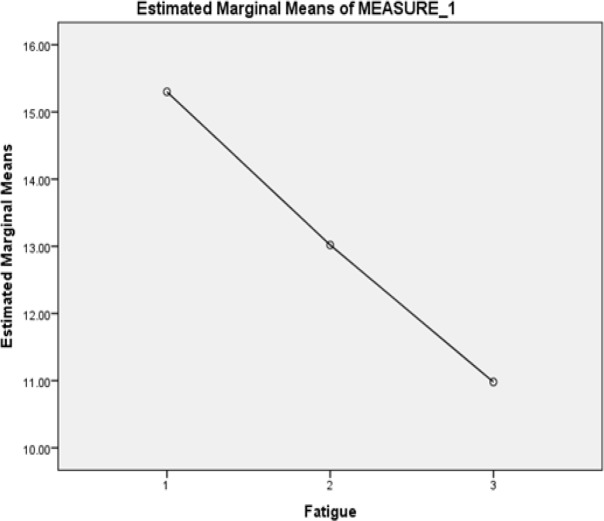
The Pattern of CRF Changes in the Patients with AML Undergoing Chemotherapy

The data analysis showed statistically significant differences in the reduction of the CRF before, on the fifth day and on the tenth day of the intervention. The mean of the CRF intensity in the last 24 hours before the program were compared with those on the fifth day and tenth days after the intervention (p<0.001) indicating a reduction of CRF in both days after the intervention (Table 3, Figure 1).

Also, the assessment of the interference of the last 24 overall CRF with patients’ daily life activities indicated that overall CRF was decreased significantly on the tenth day compared to before the intervention and on the fifth day of the intervention (p<0.001) (Table 4).

**Table 1:** The use of the Bandura’s self-efficacy theory in the intervention

Sources of self-efficacy	Methods	Descriptions
*Enactive mastery experiences*: It serves as the indicator of ability.	-Monitoring the heartbeat; -Holding weekly sessions with patients; -Making phone calls to patients	. Description of walking experiences by patients; . Reinforcing positive experiences and minimizing negative ones; . Monitoring the heartbeat . Holding sessions with patients and asking them to express their success to reach goals; . Asking patients to document their success on a daily basis so that they can monitor their own success within 10 days; . Making phone calls to patients and asking them to express their feelings and encouraging them to continue walking exercise; . Observing the success trend in the walking program by patients.
*Verbal persuasion*: It encourages the possession of certain abilities.	Providing information to patients; Providing brief recommendations for patients using the face to face method	. Verbal enhancement of the good functioning in the exercise program; . Explaining the walking program in written and verbally consisting of warming up, gradual increase of intensity, frequency and period over time; . Encouraging patients regularly.
*Vicarious experiences*: They alter self-efficacy through transmission of competencies and comparison with the achievements of others, which are provided by social role models.	Story telling/role modeling	. Storytelling of various cancer cases, for example, the story of a patient with leukemia who, by doing the exercise experienced a better quality of life and less physical, social, and psychological symptoms; . Expressing the benefits of exercise.
*Emotional/physical arousal*: Relying partly on physiological and affective statues for judging abilities.	Making phone calls Holding sessions	. Having a friendly relationship with patients and making phone calls and encouraging them to express their emotional and physical conditions; . Answering patients questions and providing adequate information about leukemia and the benefits of exercise to reduce their symptoms.

**Table 2:** The Demographic Characteristics of the Patients

Variable		N	%
Gender	Male	30	60
	Female	20	40
Marital status	Married	25	50
	Single	25	50
	Illiterate	6	12
	Elementary	9	18
	Guidance school and high school	12	24
Education level			
	Diploma	16	32
	Higher than diploma	7	14
Occupation	Self-employed	23	46
	Housewife	15	30
	Unemployed	12	24
Variable	Mean	Min	Max
Age (y)	37±12.3	18	60
Number of chemotherapy sessions	5	2	15
Duration of the disease (week)	34	18	60

**Table 3:** Comparison of the Mean Score of CRF, the Worst CRF and Overall CRF in the Last 24 Hours

Time	Before the intervention	The fifth day of the intervention	The tenth day of the intervention	P value
Variable	Mean ± Standard deviation			
Fatigue now	7.5±1.34	6.3±1.4	4.8±1.5	
Usual fatigue in the last 24 hours	7.2±1.15	6.16±1.16	5.84±1.34	<0.0001
Worst fatigue in the last 24 hours	8.4±1.22	6.86±1.34	5.84±1.34	

**Table 4:** The Comparison of the Overall 24-hour CRF Interference in Daily Life Activities

Mean of 24-hour CRF interference in the daily life activities	Mean ± Standard deviation	P value
Before the intervention	41±7.3	<0.001
on the 5^th^ day of the intervention	34.5±5.9	
on the 10^th^ day of the intervention	28.5±6.04	

## Discussion

The results of this study indicated a reduction of current CRF intensity and overall CRF interference with daily life activities including general activities, mood, normal work, walking ability, relationships with other peoples and enjoyment of life, in the last 24 hours in the patients with AML after the intervention. 

Similar studies showed the positive effects of exercise on fatigue among patients with acute leukemia undergoing chemotherapy (Chang et al., 2008; Alibhai et al., 2012; Jarden et al., 2013). Given the lack of studies on the effects of the walking exercise program on patients with AML, the results of the current study were compared with those of other studies on the exercise program in patients with other types of cancer. Studies on patients with other hematological cancers such as myelodysplastic syndrome and bone marrow transplantation showed that the exercise program had a positive effect on the reduction of CRF (Wiskemann et al., 2011; Schuler. 2016). Conversely, a study on the effect of exercise on fatigue in patients with myeloma undergoing chemotherapy and treatment with Epoetin Alfa showed no significant reduction in fatigue (Coleman et al., 2008). However, it was expected that exercise along with Epoetin Alfa would improve blood cell counts and anemia, which affected CRF. Such differences in results could be due to the type of chemotherapy regimen, nature and physiology of the different types of cancer, duration of disease and time duration of the exercise program.

Additionally, the results of some studies in other patients with other types of cancer such as breast, prostate, lung and colorectal cancers supported the positive effect of exercise on fatigue, mood disorder and walking ability (Schneider et al., 2007; Wang et al., 2011; Cheville et al., 2013). In general, CRF in patients is a common and important problem with devastating effects on all aspects of quality of life, particularly physical health, work, participation in social activities and even self-care activities such as eating and bathing. Since the diagnosis of cancer leads to fear and anxiety in such patients, it is the responsibility of healthcare staff especially nurses to improve patients’ quality of life through taking necessary measures to reduce their fatigue.

*Study limitations*

While the walking exercise program had a positive effect on the reduction of CRF, the small sample size with the limited age range and the fact that the study was limited to one hospital might have affected the generalizability of the findings to all patients with AML. Moreover, due to practical considerations no control group could be recruited. Also, the side effects of drugs, physiological issues, anemia, depression and sleep disorders might have affected the severity of CRF as confounding factors in the present study, which were not in the control of the researchers. Therefore, further research with a larger sample size and a control group with different age groups along with restricting confounding variables are suggested to improve the power of data analysis. 

In conclusion, in this study within one diagnostic group with a limited sample size that was indeed a convenience sample, the planned walking intervention seems to be an effective strategy for reducing cancer related fatigue in some patients. Therefore, it is suggested that the planned walking intervention is included in the routine nursing care of patients with AML undergoing chemotherapy. 
